# Sericin/Human Placenta-Derived Extracellular Matrix Scaffolds for Cutaneous Wound Treatment—Preparation, Characterization, *In Vitro* and *In Vivo* Analyses

**DOI:** 10.3390/pharmaceutics15020362

**Published:** 2023-01-20

**Authors:** Jayavardhini Bhoopathy, Sankari Dharmalingam, Weslen Vedakumari Sathyaraj, Selvarajan Rajendran, Shibormi Rymbai, Rethinam Senthil, Raji Atchudan

**Affiliations:** 1 Faculty of Allied Health Sciences, Chettinad Hospital and Research Institute, Chettinad Academy of Research and Education, Kelambakkam 603103, Tamil Nadu, India; 2 Department of Biotechnology, College of Science and Humanities, SRM Institute of Science and Technology, Kattankulathur 603203, Tamil Nadu, India; 3 Centre for Nano Science and Technology, Alagappa College of Technology Campus, Anna University, Chennai 600036, Tamil Nadu, India; 4 Department of Leather Engineering, Faculty of Engineering, Ege University, 35100 Izmir, Turkey; 5 School of Chemical Engineering, Yeungnam University, Gyeongsan 38541, Republic of Korea; 6 Department of Chemistry, Saveetha School of Engineering, Saveetha Institute of Medical and Technical Sciences, Chennai 602105, Tamil Nadu, India

**Keywords:** sericin, decellularized extracellular matrix, scaffolds, open excision wound

## Abstract

Human placenta is loaded with an enormous amount of endogenous growth factors, thereby making it a superior biomaterial for tissue regeneration. Sericin is a naturally occurring silk protein that is extensively used for biomedical applications. In the present work, sericin and human placenta-derived extracellular matrix were blended and fabricated in the form of scaffolds using the freeze-drying method for cutaneous wound treatment. The prepared sericin/placenta-derived extracellular matrix (SPEM) scaffolds were characterized to determine their morphology, functional groups, mechanical strength, and antibacterial activity. Scanning electron microscopic analysis of the scaffolds showed smooth surfaces with interconnected pores. *In vitro* MTT and scratch wound assays performed using HaCaT cells proved the non-toxic and wound-healing efficacy of SPEM scaffolds. *In vivo* CAM assay using fertilized chick embryos proved the angiogenic potency of the scaffolds. Animal experiments using Wistar albino rats proved that the open excision wounds treated with SPEM scaffolds significantly reduced wound size with collagen deposition. These results confirm that SPEM scaffolds can serve as a promising biomaterial for tissue regeneration.

## 1. Introduction

Skin serves as a protective interface between the host and harmful microbes/toxins and an unfavorable external environment [1]. It is the largest organ in the human body and is made up of multiple layers, such as the epidermis, dermis, and subcutaneous fat. Deep injuries continue to be a global problem among the elderly population due to health complications such as diabetes and obesity [2]. The normal structure of human skin is disrupted by a cut/abrasion, followed by the activation of a wound healing cascade comprising various cellular and molecular events to repair the injured tissues [3]. Wound healing involves four phases, hemostasis, inflammation, cellular proliferation, and remodeling [4,5]. These stages have unique biological and functional properties and rely on various growth factors, chemokines, and cytokines released by different cells at the wound site. Several materials, such as films, scaffolds, and nanofibers, are used as wound dressings to enhance the repair of full-thickness cutaneous wounds [4,6]. Biomaterial-based wound dressing has the advantage of providing an appropriate microenvironment for proper cell adhesion and proliferation to repair the structural and physiological characteristics of the wound [7]. It also serves as an active barrier against micro-organisms, allows gaseous exchange, and absorbs wound exudates [8,9,10]. Current research is focused on preparing novel wound dressing materials from naturally occurring resources for efficient tissue repair and regeneration. Tissue engineering research shows more attention toward the fabrication of matrices/scaffolds with high porosity and low/non-toxicity for wound dressing applications [11].

Sericin and fibroin are two different proteins that can be isolated from silk cocoons of *Bombyx mori* [12,13]. Sericin is a hydrophilic protein that can be extracted using the degumming method. It has high chemical reactivity because of hydrophilic macromolecular polar side chains. Several studies have reported the ability of sericin to enhance the proliferation of fibroblasts and stimulate cell migration by activating different signaling pathways [14,15]. Furthermore, sericin can also induce collagen production for the efficient treatment of wounds [16,17]. Sericin is a potential biomaterial in the field of tissue engineering due to its distinctive features, such as low immunogenicity, excellent biocompatibility, cell adhesive, and anti-oxidant properties [12,18,19]. Sericin can be fabricated as films and hydrogels for wound dressing applications [20]. Sericin can be used in combination with other polymers for biomedical applications due to its mechanical instability.

In recent years, extracellular matrix (ECM)-based biomaterials have served as desirable materials for tissue regeneration as they provide sufficient biological signals for the migration, proliferation, and differentiation of cells [21,22]. Human placenta is a complex organ that serves as a rich reservoir of extracellular matrix, bioactive molecules, cytokines, and growth factors, such as platelet-derived growth factor (PDGF), vascular endothelial growth factor (VEGF), insulin-like growth factor 1 (IGF-1), fibroblast growth factor-2 (FGF-2), epidermal growth factor (EGF), and transforming growth factor beta (TGF-β). Decellularized extracellular matrix (dECM) isolated from the human placenta has shown great promise in fabricating a biological scaffold that is capable of providing a favorable environment for wound recovery [22]. Human placenta and extracellular matrix derived from the placenta are evaluated for wound healing and the induction of angiogenesis due to the presence of several growth factors [22,23,24,25,26]. Components of the extracellular matrix are combined with bioactive components to significantly aid in tissue regeneration. dECM provides excellent support to the developing tissue due to its potential mechanical properties. In the current work, sericin/placenta-derived extracellular matrix (SPEM) scaffolds were developed by combining natural products—sericin and human placenta-derived extracellular matrix. The prepared scaffolds were characterized for physical and chemical properties. The antibacterial activity of the SPEM scaffolds was studied using the bacterial strain—*Escherichia coli*. *In vitro* experiments were carried out using human skin keratinocyte (HaCaT) cells to analyze the biocompatibility of the prepared scaffolds. The wound healing efficacy of the SPEM scaffolds was studied using a Wistar rat model.

## 2. Materials and Methods

Silk cocoons were obtained from the Government Silk Cocoon market, Vellore, India. DNase, RNase, phosphate buffered saline, sodium dodecyl sulfate, Dulbecco’s modified eagle’s medium (DMEM), trypsin—EDTA solution, fetal bovine serum, antibiotic antimycotic solution, 3-(4,5-dimethylthiazol-2-yl)-2,5-diphenyltetrazolium bromide, dimethyl sulphoxide, Luria bertani broth, nutrient agar, and formalin, were purchased from HiMedia Laboratories Private Limited, India. *Escherichia coli* was procured from Microbial Type Culture Collection, Institute of Microbial Technology, Chandigarh, India.

### 2.1. Isolation of Sericin

**Figure 1:**
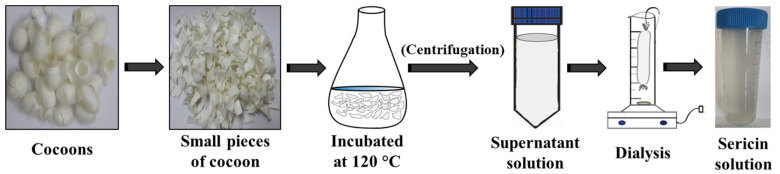
Isolation of sericin from silk cocoons—a schematic representation.

*Bombyx mori* silk cocoons were used to isolate sericin using the method described by Lamboni et al. [14]. Cocoons were cut into small pieces, soaked in deionized water (1 g of cocoons/30 mL of deionized water), and autoclaved at 120 °C for 30 min. The extract obtained was subjected to centrifugation at 8000 rpm for 15 min to remove the fibroin fibers. The final solution was dialyzed against deionized water, and the sericin obtained was stored at −20 °C (Figure 1).

### 2.2. Decellularized Extracellular Matrix (dECM) from Human Placenta

**Figure 2:**
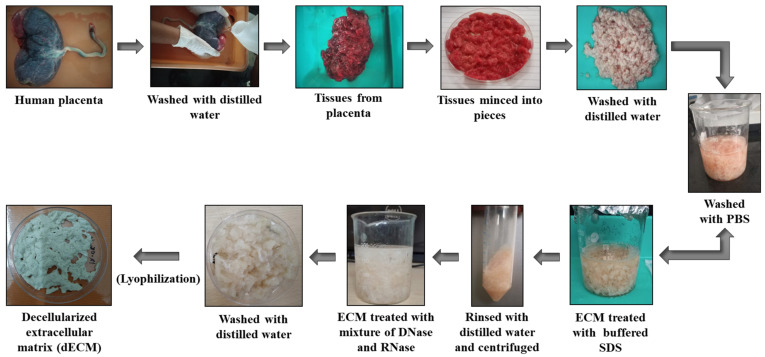
Isolation of decellularization extracellular matrix (dECM) from human placenta.

The human placenta collection was carried out as per the guidelines of the Institutional Human Ethical Committee (Proposal No. 233/IHEC/1-19) at Chettinad Academy of Research and Education, India. Placenta was isolated from individuals undergoing cesarean section (Chettinad Health City) with proper concern. The decellularized extracellular matrix (dECM) was isolated from the human placenta using the method described by Choi et al. [27]. The placenta was rinsed multiple times with deionized water to eliminate blood and impurities. The amniotic membrane was discarded, and the placental tissues were minced into small pieces using sterile scissors. The minced placenta was rinsed multiple times with deionized water to get rid of blood residues. The tissue samples were centrifuged at 8000 rpm for 10 min, and the upper layer containing blood residues was removed. The above procedure was repeated several times until the blood components were removed from the tissues. The obtained dECM was washed repeatedly with deionized water and centrifuged at 3000 rpm for 10 min. The dECM was treated with phosphate-buffered saline (PBS) and buffered 0.5% sodium dodecyl sulfate (SDS) for 30 min in a shaking water bath. The dECM treated with SDS was centrifuged and washed with distilled water several times at room temperature to remove the SDS residue. The dECM suspension was then treated with 0.2% DNase and 200 mg/mL RNase at room temperature for 10 min. The final solution was centrifuged at 8000 rpm for 10 min and rinsed thoroughly several times with deionized water. The obtained dECM was frozen and freeze-dried using lyophilization (Figure 2).

### 2.3. Preparation of Sericin/Placenta-Derived Extracellular Matrix (SPEM) Scaffolds

To 2 g of aqueous sericin solution, 1 g of dECM was added and homogenized at high speed for 30 min. The mixture was poured gently into sterile Petri plates, frozen at –80 °C, and lyophilized to obtain sericin/placenta-derived extracellular matrix (SPEM) scaffolds.

### 2.4. Characterization

A Shimadzu UV-visible spectrophotometer was used for UV spectroscopic analysis of the SPEM scaffold. FTIR characterization was performed using a Bruker Alpha FTIR spectrometer with a wavenumber resolution of 1 cm^−1^. A piece of SPEM scaffold was cut, ground well with KBr, and pressed to form pellets. The pellet was mounted into the FTIR spectrometer to obtain the functional groups in the range of 4000–500 cm^−1^. Scanning electron microscope (SEM) was used to examine the morphology of the SPEM scaffold. The scaffold was gold palladium sputtered using an Emitech SC7620 sputter coater (Quorum Technologies Ltd, Ashford, Kent, UK) and mounted onto the sample holder to view the structure using Carl Zeiss MA15/EVO 18 SEM (Carl Zeiss Microscopy GmbH Carl-Zeiss-Promenade 10 07745, Jena, Germany). The mechanical strength of the SPEM scaffold was determined using a Universal testing machine (Make: Associated Scientific Engg. Works, New Delhi, India). The gear rotation speed of the instrument is 0.5 to 100 mm/min, and the software used is FIE make India. Four samples were cut into the shape of dog bones and used for the tensile strength analysis.

### 2.5. Hemolytic Assay

Hemolysis is a process that occurs due to the impairment of red blood cells (RBC) and releases their content into the blood plasma [28]. In the present work, the hemocompatibility of the SPEM scaffolds was assessed using a hemolytic assay. A total of 10 mL of human blood was withdrawn with the consent of a healthy individual (IHEC Proposal no:233/IHEC/1-19) and centrifuged at 3000 rpm for 5 min. The pellet containing RBC was diluted in PBS. SPEM scaffolds were cut into the size of 1 × 1 cm and incubated in RBC solution for 60 min at room temperature. Water served as the positive control, and PBS as the negative control [29]. The experiment was carried out using triplicate samples. Following incubation, the samples were subjected to centrifugation at 2500 rpm for 5 min, and the supernatant was used to measure the optical density at 540 nm. The following equation was used to calculate hemolysis.

$$\mathrm{Percentage}\;\mathrm{of}\;\mathrm{hemolysis}\;\left(\%\right)=\frac{\left(\mathrm{OD}\;\mathrm{of}\;\mathrm{test}\right)-\left(\mathrm{OD}\;\mathrm{of}\;\mathrm{PBS}\right)}{\left(\mathrm{OD}\;\mathrm{of}\;\mathrm{water}\right)-\left(\mathrm{OD}\;\mathrm{of}\;\mathrm{PBS}\right)}\times 100$$

### 2.6. Swelling Study

The swelling test was used to estimate the fluid absorption property of the SPEM scaffold. The scaffold was weighed and incubated in 2 mL of sterile phosphate buffer saline (PBS) solution at room temperature. The scaffolds were withdrawn from the PBS solution at 300 min, air-dried, and weighed. The swelling capacity of the scaffolds was determined using the weight gained after the incubation period. The test was carried out in triplicates. The percentage of swelling was determined using the following equation,

Swelling (%) = ((W_s_ − W_d_)/W_d_) × 100

where W_d_ and W_s_ correspond to the measured weight of dry and swollen scaffolds, respectively. Duplicate samples were used for the experiment.

### 2.7. Moisture Content

Garrido et al. method was used to determine the moisture content of the scaffold [30]. The scaffold was cut into definite sizes (1 cm^2^) and weighed. The sample was kept in a hot air oven, and a temperature of 105 °C was maintained for 1 day. After incubation, the sample was weighed, and the percentage of the moisture content was determined using the following equation

Moisture Content (%) = ((W_iw_ − W_fw_)/W_iw_) × 100

where W_iw_ and W_fw_ correspond to the initial and final weight of the scaffolds, respectively [31]. Triplicate samples were used for the experiment.

### 2.8. Antibacterial Activity

The antibacterial activity of the SPEM scaffolds against *Escherichia coli* (*E. coli*) was studied using turbidity. The scaffold was incubated in 1 × 10^6^ CFU per ml *E. coli* suspension for 1 day. Photographs were captured after incubation, and optical density was determined at 600 nm. Sterile bacterial culture medium was used as a blank, and *E. coli* culture without scaffold was used as a negative control. The following equation was used to determine the percentage of bacterial inhibition by the SPEM scaffold [32].

Bacterial Inhibition (%) = ((B_c_ − B_t_)/B_c_) × 100

where B_c_ and B_t_ correspond to OD values of control and test, respectively. 

The spread plate method was used to study the antibacterial efficacy of SPEM scaffolds. A total of 100 µL of bacterial culture was spread over the nutrient agar, and the scaffold was placed and incubated overnight at 37 °C. Photographs were taken to observe the formation of the zone of inhibition at the end of the experiment. Duplicate samples were used for the antibacterial study.

### 2.9. In Vitro Experiments

Human skin keratinocyte (HaCaT) cells were obtained from National Center for Cell Science, Pune, India. The cells were maintained in Dulbecco’s Modified Eagle Medium (DMEM) with 4.5 g glucose per liter, L-glutamine, and sodium pyruvate. The culture was supplemented with 10% fetal bovine serum (FBS) and 1% antibiotic antimycotic solution containing penicillin, streptomycin, and amphotericin B, respectively. The cells were incubated in a HERAcell CO_2_ incubator with 5% CO_2_ at 37 °C. Accuscope inverted microscope was used for capturing photographs of the cells.

#### 2.9.1. Cell Viability Assay

Human Skin keratinocyte (HaCaT) cells were used to study the non-cytotoxic behavior of the SPEM scaffold. The cells were seeded in a 96-cell plate and allowed to proliferate for 24 h. Various dilutions of the SPEM scaffold were used for the study. Cell viability assay was performed using MTT reagents after 24 h of incubation. For this analysis, cells were treated with MTT solution for 4 h in a dark environment after treatment. Later, formazan crystals were dissolved by adding dimethyl sulfoxide, and the optical density was determined at 570 nm [31]. Triplicate samples were used for the study.

#### 2.9.2. Wound Healing Assay

HaCaT cells were seeded on 24-well plates and allowed to grow until 90% confluency was reached. Using a sterile micropipette tip, a wound was scratched, and the cells were washed with sterile PBS to eliminate dead cells and debris. A SPEM scaffold was added, and photographs were taken at different time intervals to determine the wound-healing effect of SPEM scaffolds on skin keratinocytes. Quadruplicate samples were used for the study.

### 2.10. In Vivo Experiments

#### 2.10.1. Chick Embryo Chorioallantoic Membrane Assay

An *in vivo* CAM assay was performed to evaluate the biocompatible and angiogenic potencies of the SPEM scaffold [33]. Fertilized eggs (Day 4) were purchased from the Government Poultry Farm, Kattupakkam, Tamil Nadu, India. Before starting the experiment, the eggs were cleaned with ethanol. Using sterile forceps, a small opening was created on the top of the eggshell, and the chick embryos were incubated with the SPEM scaffold. Photographs were taken at 0 and 4 h of incubation using a One Plus 10R mobile camera (OPPO Mobiles India Private Limited, Noida, Uttar Pradesh, India).

#### 2.10.2. Open Excision Wound Animal Model

**Figure 3:**
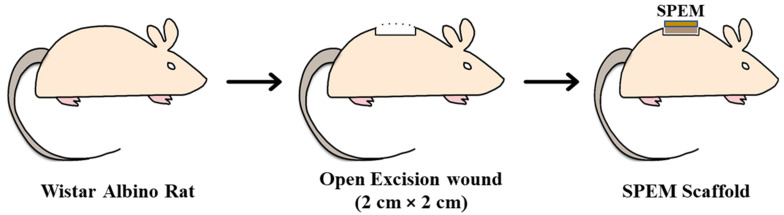
Treatment of open excision wounds using SPEM scaffold in Wistar albino rats.

Animal experiments were carried out after receiving approval from the Institutional Animal Ethics Committee (Proposal no IAEC/Proposal:31/A. Lr:13/Dt:20.12.18), Chettinad Academy of Research and Education. Male Wistar albino rats weighing 150 g were used for the wound healing experiment. Animals were housed in hygienic conditions and fed pellet food and water. Animals were anesthetized, and hair at the dorsal thoracic-lumber region was removed. Wounds measuring 2 × 2 cm were created under sterile conditions using scalpel blades and forceps. The animals were split into two different groups: Group 1—control, was treated with saline, and Group 2 was treated with SPEM, respectively (Figure 3). Both groups had five animals throughout the study.

The animals were maintained in separate cages throughout the experiment. The treatment was given once in two days, and wounds were photographed at different time periods to determine the rate of wound closure. The dimensions of the wound were measured on days 0, 5, 10, and 15. The percentage of open contraction was calculated using the formula:

$$\mathrm{Rate}\;\mathrm{of}\;\mathrm{wound}\;\mathrm{closure}\;\left(\%\right)=\frac{\;A_{o}-A_{t}}{A_{o}}\times 100$$

where *A_o_* is the initial wound size and *A_t_* is the wound size at a particular time, respectively [22]. Granulation tissues were collected from both groups on days 5, 10, and 15 and used for histological analysis. Tissues were fixed with 10% formalin, dehydrated, and embedded in paraffin. Thin sections were cut, stained with hematoxylin and eosin, and viewed under a microscope. 

### 2.11. Statistical Analysis

GraphPad version 5.03 (*t*-test) was used to analyze the data. *p*-values greater than 0.05 were considered to be statistically significant. 

## 3. Results and Discussion

In the current study, sericin was isolated from *Bombyx mori* silk cocoons. Human placenta was collected under a sterile environment from donors undergoing cesarean section.

A combinatorial treatment using SDS and nucleases was employed to prepare dECM from the placenta. Both sericin and dECM were combined together to fabricate a novel biopolymeric scaffold that can be used as a wound healing agent. A simple, cost-effective method has been used to prepare the SPEM scaffold for healing open excision-type wounds. In the present study, the SPEM scaffolds were stored at −20 °C for up to 30 days to maintain their bioactivity.

### 3.1. Characterization

**Figure 4:**
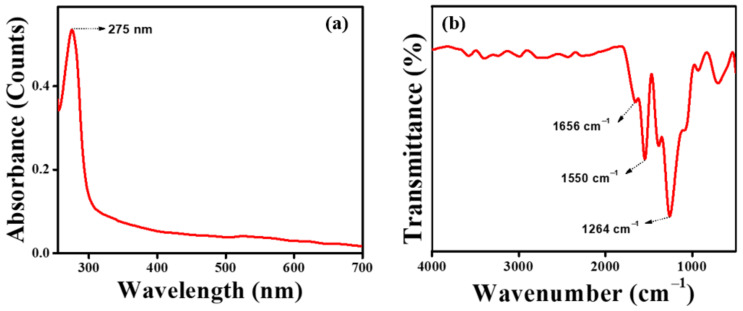
(**a**) UV-visible spectrometric measurement of the SPEM scaffold, and (**b**) FTIR spectrum of the SPEM scaffold.

**Figure 5:**
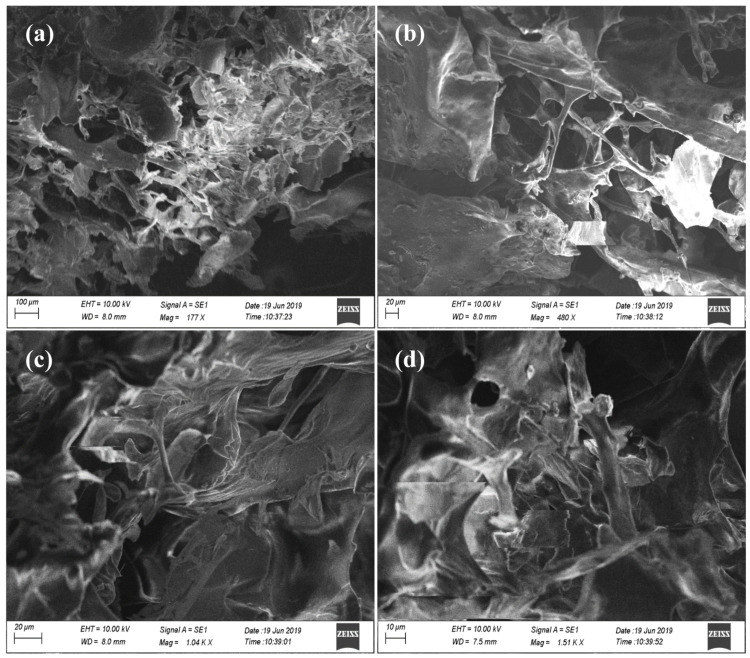
SEM images of the SPEM scaffold with different magnifications, (**a**) 100 µm, (**b**,**c**) 20 µm, and (**d**) 10 µm, showing the irregular pores with few interconnections between them.

UV-visible spectrometric (Figure 4a) measurement of the SPEM scaffold showed maximum absorption at 275 nm, confirming the presence of tyrosine amino acid in sericin. An FTIR was performed to determine the functional groups present in the SPEM scaffold. Most of the characteristic peaks of sericin and dECM were difficult to differentiate as they were merged together because of their proteinaceous nature. Figure 4b shows the characteristic absorption peaks corresponding to the peptide bonds that form amide I, amide II, and amide III bands at 1656, 1550, and 1264 cm^−1^, respectively. No significant variation or formation of new peaks was detected in the FTIR spectrum of the SPEM scaffold. SEM was used to examine the microstructure of SPEM to determine its structure and homogeneity. Figure 5 shows the SEM microstructure of the SPEM scaffold containing irregular pores with few interconnections between them. The addition of dECM to sericin has altered the microstructure of the scaffolds and resulted in irregular morphology. The wound dressing material should retain good mechanical strength during handling because, when applied to the wound site, it should be stable without any rupturing. The scaffold used as a wound dressing must be rigid and firm throughout the healing process. In the present study, an Instron universal testing machine was used to determine the mechanical properties of the prepared scaffold. The results showed that the SPEM scaffold had a tensile strength of 0.154 ± 0.051 MPa. The young’s modulus for the SPEM scaffold is calculated as 0.011 MPa. Mechanical strength is crucial for wound dressing material as it must withstand high blood flow and prevent fragmentation. The SPEM scaffold can be used as efficient wound dressing material as it holds the required strength to adhere to the wound and elicit the process of wound healing.

### 3.2. Hemolytic Assay

**Figure 6:**
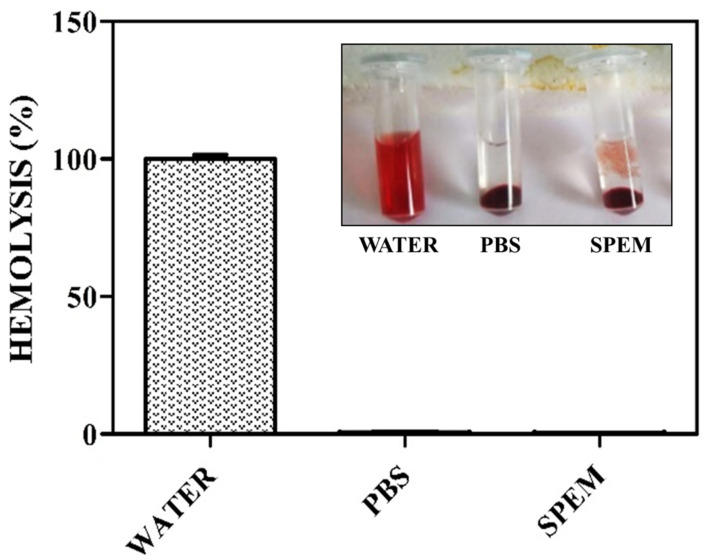
Hemolytic assay of the SPEM scaffold. PBS and water were used as negative and positive controls. Inset image shows pictures of RBC treated with water, PBS, and SPEM scaffold, respectively.

In order to determine the non-toxic property of the prepared SPEM scaffold, the hemolytic assay was performed using human red blood cells (RBC). When a foreign body interacts with the RBC, it might cause damage to the cell membrane resulting in leakage of hemoglobin [29]. As per the rules of the American society for testing and materials, a biomaterial with hemolysis of less than 2% is considered to be hemocompatible. Figure 6 shows 0% hemolysis for negative control and 100% for positive control. When compared with control groups, SPEM scaffolds exhibited a percentage of hemolysis less than 0.5. Hence the prepared scaffold proved to be hemocompatible and can be used as a suitable matrix for tissue regeneration.

### 3.3. Swelling Assay

The swelling test was performed for the prepared scaffolds to estimate their fluid uptake ability. Swelling behavior is one of the crucial features of wound dressing materials, as they must absorb blood and wound exudates when applied to the wound. The swelling assay is carried out to determine the percentage of liquid and nutrient absorption/transport within the scaffolds [34]. It is also used to evaluate biological fixation to the wound bed. In the present study, the swelling rate increased after incubation in PBS with the swelling ratio of SPEM at 300 min as 942.08%. The freeze-drying method employed to prepare the scaffolds results in the evaporation of water to gas molecules, leaving behind a larger pore volume, which has increased the water absorption property of the scaffold.

### 3.4. Moisture Content

Moisture content of the scaffold is another important criteria for an ideal wound dressing material. In the present study, the SPEM scaffold exhibits the percentage of moisture content as 32.38%. The result obtained represents the change in the percentage of the SPEM scaffolds after incubating in the appropriate atmosphere.

### 3.5. Antibacterial Activity

**Figure 7:**
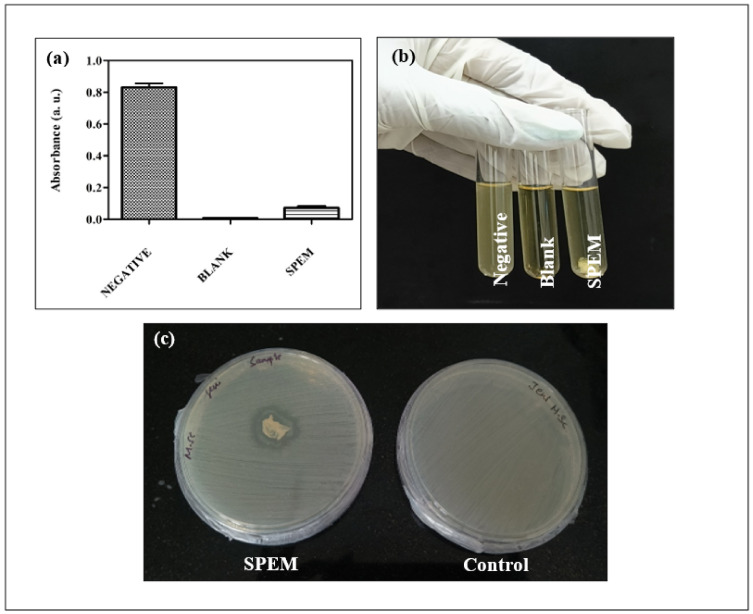
Turbidity analysis: (**a**) Turbidity analysis showing the optical density of the negative control, blank, and SPEM scaffold after incubation (**b**) Pictures showing negative control, blank, and *E. coli* culture incubated overnight with SPEM scaffold. Full growth was seen in the negative control, whereas no growth was seen in the blank. Spread plate method: (**c**) Pictures showing the zone of inhibition formed after treatment with the SPEM scaffold.

Microbial infections are considered significant threats during the course of wound treatment. They can also cause life-threatening infections [35]. A perfect wound dressing material must hold efficient antibacterial properties to overcome the disease caused by the microbes that are associated with wound infections. Turbidity analysis showed the percentage of bacterial inhibition as 91.2% after incubation with the SPEM scaffold. Figure 7a represents the optical density of the blank, negative control, and SPEM scaffold after incubation. The tube containing the test sample showed no growth as that of the blank and appeared to be transparent (Figure 7b). Figure 7c shows the antibacterial efficacy of the SPEM scaffold against *E. coli* using the spread plate method. The zone of inhibition was formed after incubation with the SPEM scaffold. These results proved that SPEM scaffolds have antibacterial activity and hence can be used for wound dressing applications.

### 3.6. In Vitro Experiments

#### 3.6.1. Cell Viability Assay

**Figure 8:**
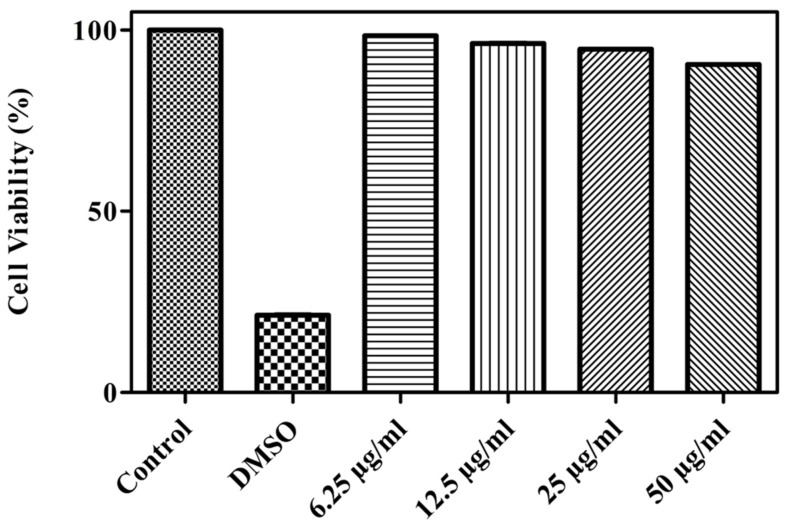
Viability of HaCaT cells incubated with different concentrations of SPEM scaffold for 24 h.

One of the characteristic features of wound dressing material is that it must enhance the survival and proliferation of cells. In the present study, the biocompatible nature of the SPEM scaffold was studied using HaCaT cells. In general, any material that comes in contact with the cellular membrane may trigger the discharge of toxic elements and cause damage to cells. MTT assay is a typical method that is commonly performed to study the cytocompatible property of biomaterials. Figure 8 shows the percentage of viable cells after incubation with different concentrations of SPEM scaffolds for 24 h. Several reports have proven the effect of sericin as an excellent biomaterial for tissue regeneration and wound treatment [36]. Sericin-based materials provide the required microenvironment for the growth and differentiation of cells. Moreover, dECM possesses numerous growth factors that are responsible for tissue formation [22]. Thus, MTT results demonstrate the non-cytotoxic behavior of prepared SPEM scaffolds and thereby confirm their use for biomedical applications.

#### 3.6.2. Wound Healing Assay

**Figure 9:**
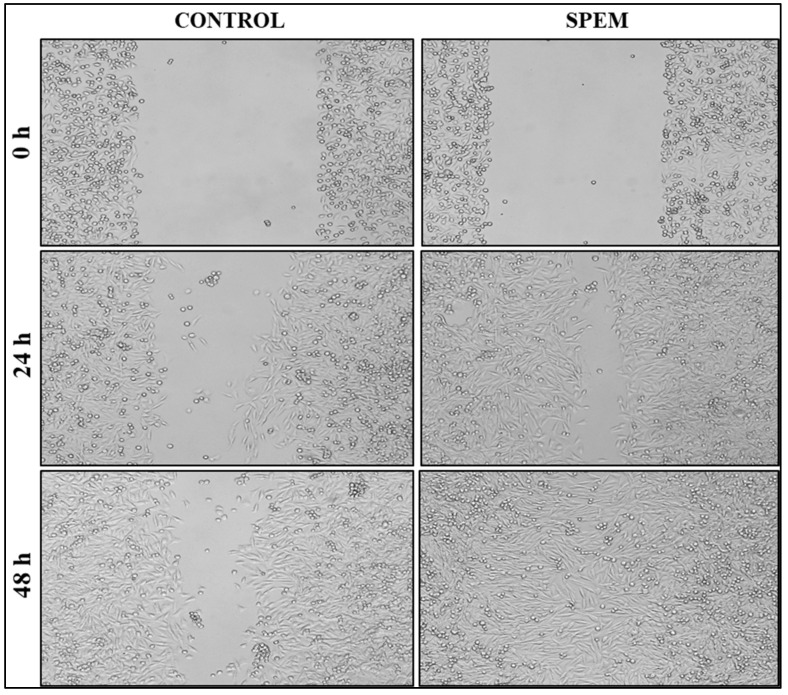
Scratch wound assay using SPEM scaffolds. Wound closure of 98% was observed after 48 h of treatment with SPEM scaffold (Magnification: 4× objective).

**Figure 10:**
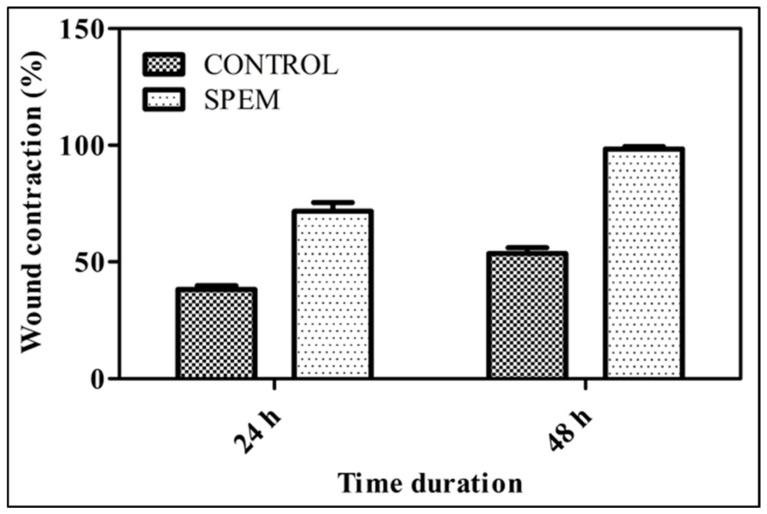
Percentage of wound closure in SPEM-treated and untreated cells for 24 and 48 h, respectively.

*In vitro* scratch wound assay was performed to determine the wound healing efficacy of SPEM scaffolds. Figure 9 shows the microscopic images of HaCaT cells before and after treatment. Significant wound closure was observed within 24 h of incubation compared to the untreated control. Wound closure of 72% and 98% was observed after 24 and 48 h of treatment, whereas untreated control cells showed only 38% and 54%, respectively (Figure 10). These results prove that the prepared SPEM scaffold can efficiently heal wounds by enhancing the migration of cells, but *in vivo* experiments must be performed to substantiate the *in vitro* result obtained. 

### 3.7. In-Vivo Experiments

#### 3.7.1. Chick Embryo Chorioallantoic Membrane Assay

**Figure 11:**
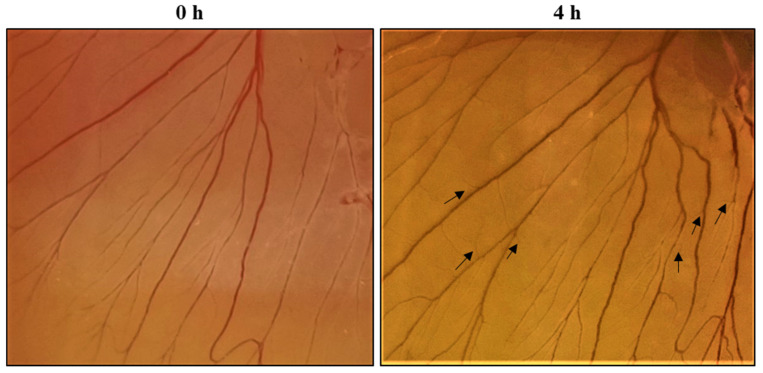
Photographs of *in vivo* CAM assay in the presence of SPEM scaffold. Black arrows indicate the new blood vessels formed after 4 h of treatment.

Angiogenesis is the process of the formation of new blood vessels from previously existing ones. Blood vessels are formed either by sprouting or branching processes. Angiogenesis is vital for wound healing because the newly formed blood vessels are involved in supplying nutrients and oxygen to the developing tissues [37]. CAM Assay is one of the most accepted standard protocols to study the angiogenic potency of any material. In the present study, chick embryos treated with SPEM scaffolds showed the formation of new blood vessels after 4 h of incubation. In Figure 11, the black arrows indicate the new blood vessels formed after treatment. Mobile camera with 50 MP was used for capturing the images. It is proposed that sericin can increase the expression of tumor necrosis factor-alpha (TNF-α), which, in turn, serves as an activator of hypoxia-inducible factor (HIF). HIF can elevate the expression of vascular endothelial growth factor (VEGF). Moreover, dECM is loaded with various growth factors that could have resulted in angiogenesis.

#### 3.7.2. Open Excision Wound Animal Model

**Figure 12:**
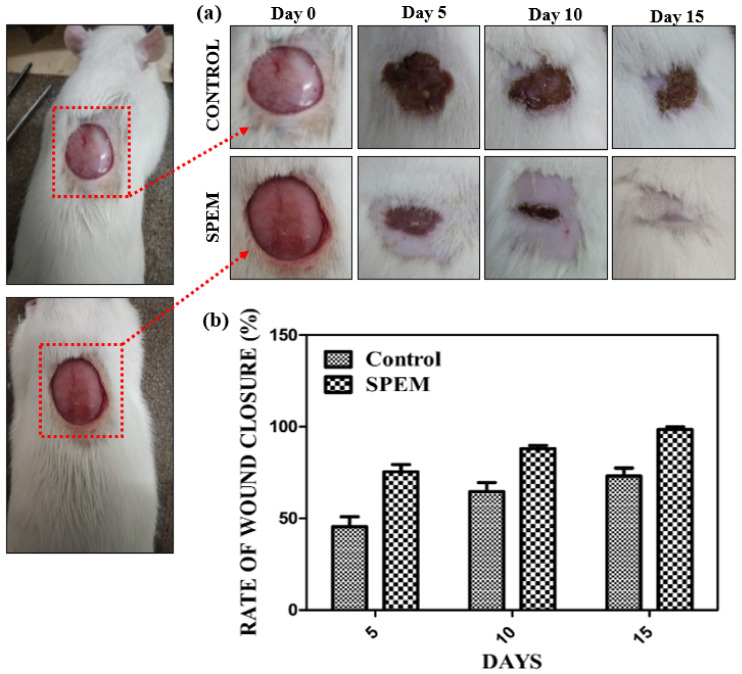
(**a**) *In vivo* wound healing images of control and SPEM-treated groups on days 5, 10, and 15, respectively. (**b**) Percentage of wound closure in open excision rat wound model.

Based on the hemocompatibility and *in vitro* cytocompatibility of the prepared SPEM scaffolds, an *in vivo* animal model was used for further experiments. The open excision wound rat model was used to determine the wound healing property of the SPEM scaffold. The control was treated with saline, and the test group was treated with the SPEM scaffold. The photographs taken on specific days from day 0 showed a significant reduction in wound size in groups treated with the SPEM scaffold compared with the control (Figure 12a). It was also observed that when applied to the wounded site, SPEM scaffolds were readily attached to the surface, which is one of the ideal properties of wound dressing materials. The percentage of wound contraction in SPEM-treated groups was determined as 75%, 88%, and 98% on days 5, 10, and 15, respectively, which was significantly higher than the control. Wounds treated with the SPEM scaffold showed complete wound closure on day 15, whereas the control group took 20 days to completely heal the open excision wound (Figure 12b). The faster wound healing rate might be attributed to various growth factors in dECM and to the sericin’s property of inducing the proliferation of fibroblasts, keratinocytes, and other cells that play an important role in the faster healing of wounds.

#### 3.7.3. Histological Observation

**Figure 13:**
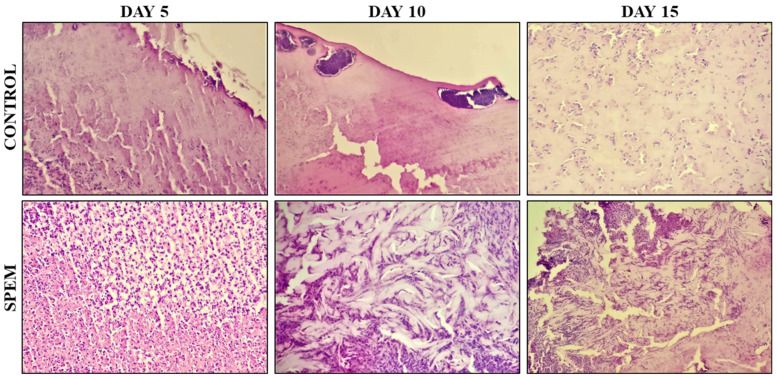
Histological analysis of the granulation tissues collected on days 5, 10, and 15 from control and SPEM-treated groups. 10× magnification was used for hematoxylin and eosin staining analysis.

Histological evaluation was performed to study the healing pattern of wounds in the control and test group (SPEM). On day 5, excision wounds treated with saline (control) showed a squamous epithelium with ulceration and necrotic keratinocytes. The group treated with SPEM showed extensive ulceration with neutrophilic infiltrate and karyorrhectic debris. On day 10, excision wounds treated with saline showed ulceration, neutrophilic infiltrate, and many bacterial colonies. The group treated with SPEM showed ulceration, and dense nan neutrophilic infiltrate with increased dermal collagen, but few capillaries were found (not well-formed granulation tissue). On day 15, wounds treated with saline showed ulceration with marked neutrophilic infiltrate, edema, and many necrotic keratinocytes. No granulation tissue or collagen was seen. The group treated with SPEM showed hyperplasia of the epidermis, dense neutrophil infiltrates with granulation tissue, and increased collagen deposition. As shown in Figure 13, the SPEM-treated group showed a significant increase in collagen formation on days 5, 10, and 15 compared to the control. The presence of collagen deposition in SPEM-treated rats has increased their healing period at the wound site. From these observations, SPEM proves that it could significantly increase the wound healing process.

## 4. Conclusions

A cost-effective method has been developed for the preparation of SPEM scaffolds using sericin and decellularized extracellular matrix. Sericin and human placenta-derived extracellular matrix were blended and fabricated in the form of scaffolds using the freeze-drying method. SEM analysis showed irregular pores with few interconnections. The swelling behavior of the prepared SPEM scaffold showed significant liquid absorption capacity with a maximum swelling of 942.08% at 300 min. Hemolytic assay results demonstrated that the SPEM scaffold did not exhibit any toxic response when treated with human RBC. Antibacterial activity of the SPEM scaffold was studied using *E. coli* cells. Scratch wound assay using HaCaT cells showed 98% wound closure after 48 h of SPEM treatment. An *in vivo* rat wound healing model proved that the SPEM scaffold, when applied to open excision wounds, showed a faster rate of wound healing when compared with untreated control. Thus, from these observations, it is suggested that SPEM scaffold can serve as a potential material for wound healing applications. 

## Data Availability

Not applicable.
